# Application of MRI for the Diagnosis of Neoplasms

**DOI:** 10.1155/2018/2715831

**Published:** 2018-02-11

**Authors:** Ewa Bejer-Oleńska, Michael Thoene, Andrzej Włodarczyk, Joanna Wojtkiewicz

**Affiliations:** ^1^Department of Pathophysiology, School of Medicine, Collegium Medicum, University of Warmia and Mazury in Olsztyn, Olsztyn, Poland; ^2^Department of Medical Biology, School of Medicine, Collegium Medicum, University of Warmia and Mazury in Olsztyn, Olsztyn, Poland; ^3^Department of Public Health and Epidemiology, School of Medicine, Collegium Medicum, University of Warmia and Mazury in Olsztyn, Olsztyn, Poland; ^4^Laboratory of Regenerative Medicine, University of Warmia and Mazury in Olsztyn, Olsztyn, Poland

## Abstract

**Aim:**

The aim of the study was to determine the most commonly diagnosed neoplasms in the MRI scanned patient population and indicate correlations based on the descriptive variables.

**Methods:**

The SPSS software was used to determine the incidence of neoplasms within the specific diagnoses based on the descriptive variables of the studied population. Over a five year period, 791 patients and 839 MRI scans were identified in neoplasm category (C00-D48 according to the International Statistical Classification of Diseases and Related Health Problems ICD-10).

**Results:**

More women (56%) than men (44%) represented C00-D48. Three categories of neoplasms were recorded. Furthermore, benign neoplasms were the most numerous, diagnosed mainly in patients in the fifth decade of life, and included benign neoplasms of the brain and other parts of the central nervous system.

**Conclusions:**

Males ≤ 30 years of age with neoplasms had three times higher MRI scans rate than females of the same age group; even though females had much higher scans rate in every other category. The young males are more often selected for these scans if a neoplasm is suspected. Finally, the number of MRI-diagnosed neoplasms showed a linear annual increase.

## 1. Introduction

Magnetic Resonance Imaging (MRI) can generate mass amounts of data within a narrow time frame. Furthermore, it is safer than and not as invasive as other imaging techniques [1]. The most popular areas for examination have been the spine and head, over the last ten years. In fact, more than half of the MRI scans have been concentrated on these areas of the body [2]. Disorders of the central nervous system, backbone, and spine have been commonly analyzed with this technique, as well as cardiovascular system diseases and disorders of the extremities [3, 4]. At emergency departments, scans are performed for head diagnostics in nearly half of all cases [5]. The literature points to the main advantages of MRI imaging techniques being related to scans of the head and neck, the chest and breast areas, the abdomen and pelvis, and the musculoskeletal system. This technique has been used in prenatal diagnostics as well [6].

MRI shows more anatomic details of the nervous system and then other techniques such as computed tomography (CT), which is especially important in discovering neoplasms of the central nervous system. This technique is particularly useful in diagnoses of the cerebellopontine angle, for diagnosing vestibulocochlear nerve tumours, as well as investigating other lesions localized in the posterior cranial fossa [7].

According to statistical data regarding the main health problems in Poland, cardiovascular diseases are the first cause of death, and cancers are the second cause of death. In the category of malignant neoplasms, the most frequently occurring were observed cancerof the lung (C34), stomach (C16), large intestine (C18, C20, C19, and C21), pancreas (C25), urinary bladder (C67), larynx (C32), prostatic gland (C61) in males, and uterus (C54, C53) or mammary gland (C50) in females [8–10]. Cancer is a worldwide health problem. Among member countries of the Organization for Economic Cooperation and Development (OECD), cancers are the second leading cause of death. There are more than 100 different types of cancers and the risk of developing many of them increases with age. Generally in OECD countries, the cancer incidence was higher for men than for women. The statistics of cancer incidence indicate that the five most common cancers in males are the prostate, lung and bronchus, colon and rectum, urinary bladder, and melanoma of the skin, whereas the most common cancers in females are found to be in the breast, lung, and bronchus, colon and rectum, and uterine corpus or in the thyroid gland [11, 12].

The aim of the present study was to determine the most commonly diagnosed neoplasms in the MRI scanned patient population and indicate correlations based on the descriptive variables.

## 2. Materials and Methods

The study population consisted of patients examined by a Siemens Magnetom Trio A Tim System at the University Clinical Hospital in Olsztyn (UCH) during the years 2011–2015. The data was collected at the MRI Laboratory of the UCH. The patient population was within the age range of 2 to 92 years. The number of scans and patients and patient characteristics were analyzed to find correlations with C and D letter-coded category according to the International Statistical Classification of Diseases and Related Health Problems (ICD-10) [13]. The patients and diagnosis coding procedure has been described in our previous publication [14].

IBM SPSS Statistics v.24 software was used for data processing and analysis. Annual data as well as data spanning a 5-year period was analyzed to determine general demographics and overall diagnostics with regard to the number of examinations performed (number of visits) or number of patients which presented C and D letter-coded category. To characterize the MRI application, descriptive statistics were used for the selected area of interest. SPSS was used to estimate variables such as the gender, age of the patient, and the specific diagnostic code. Cross-tabulations were used to check for correlation between variables, to determine the level of significance in chosen areas; the *χ*^2^ test was used, but only if each group had at least five observations.

## 3. Results

Between 2011 and 2015, there were 13,298 MRI scans conducted at the MRI Laboratory of the UCH. Analysis of a distribution of letter-coded categories according to the ICD-10 has been performed in our previous publication [14]. The most common MRI examined groups of diseases and disorders were identified, and the neoplasm category (C and D) is one of the five most often diagnosed in UCH.  Table 1 shows the number of examinations and the number of patients associated with C and D letter-coded category in 5-year period and individually in each year of the period from 2011 to 2015. Patients with C and D codes are treated as one category including all neoplasms (C00-D48), diseases of the blood and blood-forming organs and certain disorders involving the immune mechanisms (D50-D89). There were 792 patients in the C and D letter-coded category. However, one patient with a D 64 code presented a blood disease and was therefore removed from statistical considerations, since it was not a neoplasm.

Only in the subgroup of C and D under 30 years of age there were more male than female patients. Male patients were commonly MRI-diagnosed only when they were in the young group of patients ≤ 30 in the subgroup of neoplasm diagnosis. In the remaining age subgroups, the number of female patients was larger than the number of male patients. The total number of male patients in the C and D letter-coded category was lower than the total number of female patients and, respectively, amounted 44% and 56%.

In the neoplasm category (C00-D48), 108 individual diagnostic codes were recorded during the 5-year study period. 791 patients were examined and 839 MRI scans were performed. Three categories of neoplasms were recorded: malignant neoplasms (diagnosis ICD-10 codes C00-C97), benign neoplasms (codes D10-D36), neoplasms of uncertain or unknown behaviour (codes D37-D48), but there were no patients with neoplasms in situ.

**Figure 1:**
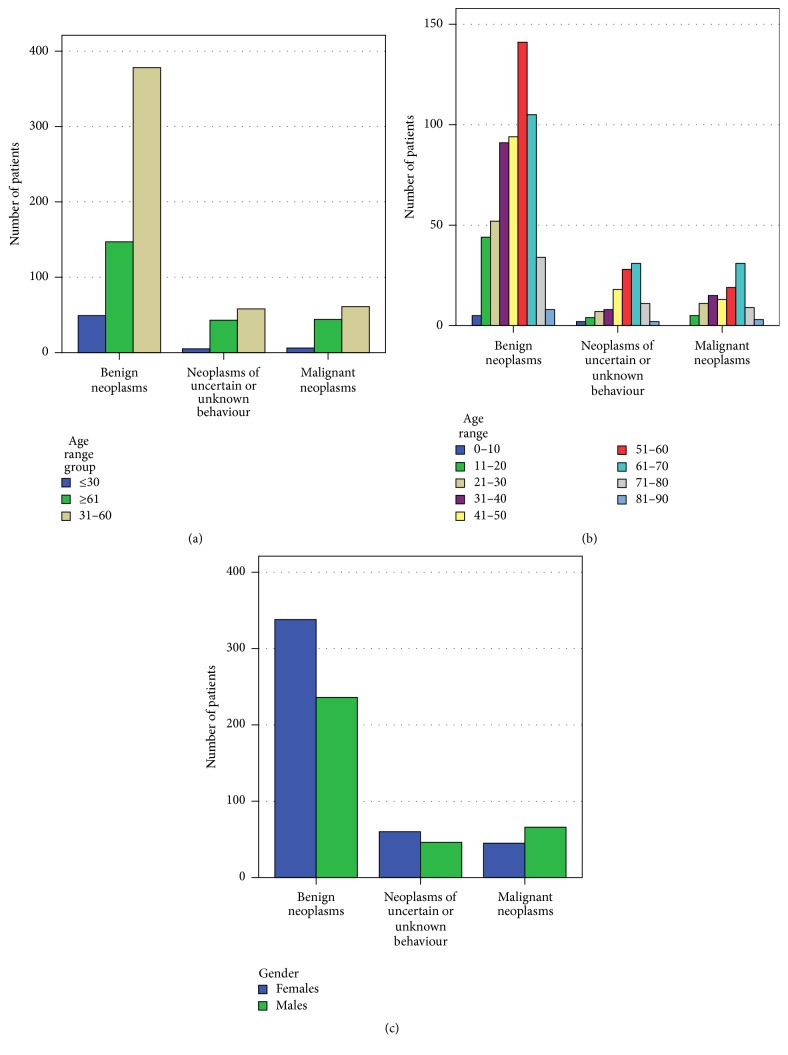
Number of patients with age range (a); 10-year intervals (b); gender (c) breakdown in relation to the three categories of neoplasms in the 5-year period of MRI utilization.

Figure 1 shows the number of patients presenting with the three types of neoplasm in the context of age and gender categories. Among all neoplasm categories, the most numerous were benign neoplasms in both female and male patients, which were diagnosed in the highest numbers in patients aged 51–60 years, whereas two other neoplasm categories dominated in patients between 61 and 70 years of age (Figure 1(b)). The malignant neoplasms were more often found in male patients in comparison to female patients (Figure 1(c)). Neoplasms were most frequently diagnosed in patients in the 31–60 age group, and relatively small numbers of neoplasms were MRI-diagnosed in patients under 30 years of age (Figure 1(a)).

**Figure 2:**
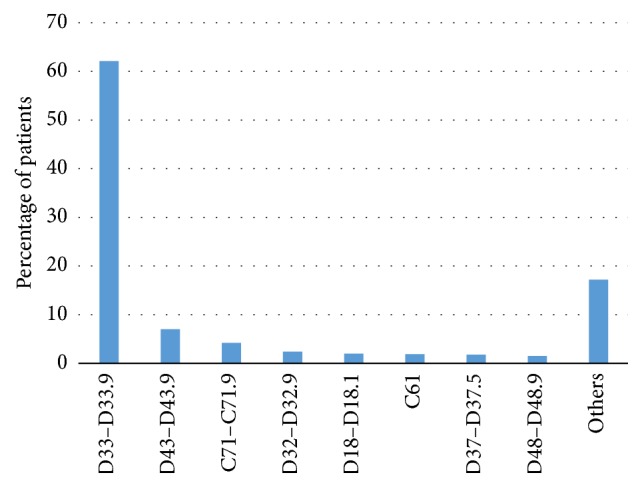
Percentage of patients with diagnosed neoplasms during 5-year MRI use. *D33*: benign neoplasm of brain and other parts of central nervous system,* D43*: neoplasm of uncertain or unknown behaviour of brain and central nervous system,* C71*: malignant neoplasm of brain,* D32*: benign neoplasm of meninges,* D18*: haemangioma and lymphangioma, any site,* C61*: malignant neoplasm of prostate,* D37*: neoplasm of uncertain or unknown behaviour of oral cavity and digestive organs, and* D48*: neoplasm of uncertain or unknown behaviour of other and unspecified sites.

The most commonly MRI-diagnosed neoplasms were benign neoplasms of the brain and other parts of the central nervous system (codes D33), neoplasms of uncertain or unknown behaviour in the brain and central nervous system (D43), malignant neoplasms of the brain (C71), and benign neoplasms of the meninges (D32). These diseases were diagnosed in 598 patients (Table 2) which constituted 75.6% of all patients with neoplasms (Figure 2). The D33, D43, and D32 letter-coded categories were dominant in females, in contrast to malignant neoplasms of the brain (C71), which were commonly diagnosed in males of all age ranges. The letter-coded categories mentioned above mostly represented patients between 31 and 60 years of age in both gender categories. Malignant neoplasms of the prostate (C61) were mainly diagnosed in males above 60 years of age (Table 2).

## 4. Discussion

There is evidence that the experience of medical practitioners coupled with timely convenient access to diagnostic equipment greatly impacts the type and frequency of diagnostic imaging. Furthermore, the use of imaging techniques varies with the symptoms and is correlated to age, gender, and any physical limitations [6].

According to previously published statistical data, the highest cancer incidence in males was in the age range of 55–74 and in the age range of 50–69 in females. Young and middle-aged females present a twice higher incidence of cancer than males [9]. Our analyses indicated a slightly earlier age range of neoplasm diagnosis (Figures 1(a) and 1(b)) and greater participation of female patients > 30 years of age in MRI method as neoplasms diagnostic tool than male patients. The MRI technique is especially useful in central nervous system tumour identification as a basic method for planning stereotactic surgery. It gives the greatest opportunity for visualizing detailed tumour characteristics, especially its vascularisation and an evaluation of metabolic intensity. Functional Magnetic Resonance Imaging (fMRI) makes it possible to provide complicated surgical intervention in an accurate way and save cortical areas. Common intracranial benign neoplasms are meningiomas (D32) which include hormonal etiopathogenesis of the meningioma, commonly occurring in females between 40 and 60 years of age [15], which is in line with data observed in our study population (Figure 2, Table 2). More than 50% of newly diagnosed cancers in Poland were in the advanced stage with little chance of successful treatment [16]. In MRI patient data, benign neoplasms dominated when compared to malignant neoplasms (Figure 1(c)). Early detection of cancer and noninvasive, early, and accurate diagnostics can reduce mortality caused by cancer, therefore MRI is used in cancer detection, staging, and monitoring and in providing small-invasive therapeutic manipulation. Unfortunately due to the cost, in some cancers the MRI technique is very rarely applied, that is, in breast cancer, where MRI detection shows higher sensitivity to invasive cancers than mammography and allows detecting cancers not identified with other types of screening [17]. In some patients from high risk-groups, the usage of this technique is certainly justifiable. However, there is no strong evidence to support the idea of an improvement in patient outcomes during routine practice [18]. That could give an explanation for no breast cancers being diagnosed in our study population.

Sun et al. (2015) [19] showed that there is strong evidence confirming gender differences in cancer prevalence and grade, especially if the localization is not strictly linked with the organs of the reproductive system. Some authors have emphasised a more common prevalence of cancer in males. Hormonal impact in malignant transformation and cancer progression in males is strongly considered. Our results are in line with these findings. We have indicated a higher incidence of malignant neoplasms of the brain in male patients as well as the total number of malignant neoplasms indicated in the examined population (Table 2, Figure 1(c)).

Prostatic gland cancer is very significant health problem in Poland and worldwide [11, 12]. Our study has shown that patients with a malignant neoplasm of the prostate (C61) participated in MRI diagnostics (Table 2).

Some authors suggested that neuroimaging methods should not be ordered for routine headaches; however, this is not true for patients with a neoplasm suspicion. Neuroimaging could save lives if it is used in order to confirm a neoplasm suspicion [20].

Unfortunately, this study only focused on one academic teaching hospital for a period of 5 years. The volume of data was not extensive, and a more detailed analysis is recommended. A more extensive analysis should be performed over a period of 10 to 15 years in order to follow the trends in MRI-diagnosed neoplasms. It is hoped that this present analysis provided enough descriptive statistics to begin describing the population at UCH that was diagnosed using MRI.

## 5. Conclusions

This analysis presented MRI-generated data and specifically identified which applications of this technique were used to describe the distribution of patients with specific neoplasm subgroups. This analysis has shown that, in the population studied, the MRI is very commonly used in the diagnoses of neoplasms, and especially neoplasms of the nervous system. Males ≤ 30 years of age with neoplasms had three times higher MRI scans rate than females of the same age group, excluding the fact that females had much higher scans rate in every other category. Furthermore, during the 5-year study period most patients presented with MRI-diagnosed benign neoplasms. These patients were in the age group of 51–60 years. Finally, the number of MRI-diagnosed neoplasms showed a linear annual increase. Therefore, the general use of MRI scans to detect neoplasms has been steadily increasing, and young males are more often selected for these scans if a neoplasm is suspected.

## Figures and Tables

**Table 1:** MRI examinations and patients during the 5-year period, 2011–2015, in C and D letter-coded category.

Letter-coded category	Visits during 5-year period				Patients										
	C		D		Females during 5-year period			Males during 5-year period			Annual number of patients				
C and D	Number	Percentage	Number	Percentage	≤30	31–60^a^	≥61	≤30	31–60^a^	≥61	2011	2012	2013	2014	2015
	118	0.9^*∗*^	722	5.4^*∗*^	26	273	145	34	224	90	103	158	170	168	193
Total	840 6.3^*∗*^				444			348							
					792						792				

*Source*. MRI Laboratory, University Clinical Hospital in Olsztyn, 2011–2015. ^*∗*^Percentage of the total number 13298 examinations performed during 5-year MRI utilization; ^a^*p* < 0.05.

**Table 2:** Neoplasms in the context of demographic characteristics of the MRI diagnosed population between 2011 and 2015.

Neoplasms	Females				Males				Total
	≤30	31–60	≥61	Total	≤30	31–60	≥61	Total	
D33–D33.9	20	180	91	291	18	148	34	200	491
D43–D43.9	0	24	13	37	1	9	8	18	55
C71–C71.9	0	10	4	14	2	12	5	19	33
D32–D32.9	0	12	0	12	0	4	3	7	19
D18–D18.1	1	5	2	8	3	5	0	8	16
C61	0	0	0	0	0	2	13	15	15
D37–D37.5	0	2	1	3	3	5	3	11	14
D48–D48.9	1	6	2	9	0	2	1	3	12
Others	4	34	31	69	7	37	23	67	136
Total	26	273	144	443	34	224	90	348	791

*Source*. MRI Laboratory, University Clinical Hospital in Olsztyn, 2011–2015.
